# High percentage of bone marrow CD8^+^ tissue-resident-like memory T cells predicts inferior survival in patients with acute myeloid leukemia

**DOI:** 10.1097/BS9.0000000000000194

**Published:** 2024-06-07

**Authors:** Letong Cai, Wenpu Lai, Danlin Yao, Yinfeng Gu, Chaofeng Liang, Lian Liu, Jing Lai, Zhi Yu, Xianfeng Zha, Xibao Yu, Xiuli Wu, Shaohua Chen, Oscar Junhong Luo, Yangqiu Li, Chunyan Wang, Pengfei Qin, Xin Huang, Ling Xu

**Affiliations:** a Key Laboratory for Regenerative Medicine of Ministry of Education, Institute of Hematology, School of Medicine, Department of Hematology, The First Affiliated Hospital Jinan University, Guangzhou 510632, China; b Department of Systems Biomedical Sciences, School of Medicine, Jinan University, Guangzhou 510632, China; c Department of Clinical Laboratory, First Affiliated Hospital, Jinan University, Guangzhou 510632, China; d Key Laboratory of Viral Pathogenesis & Infection Prevention and Control (Jinan University), Ministry of Education, Guangzhou 510632, China; e Department of Hematology, First Affiliated Hospital of Guangzhou Medical University, Guangzhou Medical University, Guangzhou, Guangdong, China; f Department of Hematology, Guangdong Provincial People’s Hospital (Guangdong Academy of Medical Sciences), Southern Medical University, Guangzhou, Guangdong, China

**Keywords:** Acute myeloid leukemia, Bone marrow, CD69, Tissue-resident-like memory T cells

## Abstract

Tissue-resident memory T (TRM) cells infiltrating solid tumors could influence tumor progression and the response to immune therapies. However, the proportion and prognostic value of TRM cells in the bone marrow (BM) of patients with acute myeloid leukemia (AML) are unclear. In this study, we used flow cytometry to assay the phenotype of 49 BM samples from patients newly diagnosed with AML (ND-AML). We found that the BM CD8^+^ effector memory (TEM) cells highly expressed CD69 (CD8^+^ TRM-like T cells), and their percentage was significantly increased in patients with ND-AML compared with that in healthy individuals (HI). The high percentage of CD8^+^ TRM-like subset was associated with poor overall survival in our ND-AML cohort. The Kaplan–Meier Plotter database verified a significantly reduced survival rate among patients with high expression of CD8^+^ TRM-like T cell characteristic genes (*CD8A*, *CD69*, and *TOX*), especially the M4 and M5 subtypes. Phenotypic analysis revealed that the BM CD8^+^ TRM-like subpopulation exhibited exhausted T cell characteristics, but its high expression of CD27 and CD28 and low expression of CD57 suggested its high proliferative potential. The single-cell proteogenomic dataset confirmed the existence of TRM-like CD8^+^ T cells in the BM of patients with AML and verified the high expression of immune checkpoints and costimulatory molecules. In conclusion, we found that the accumulation of BM CD8^+^ TRM-like cells could be an immune-related survival prediction marker for patients with AML.

## 1. INTRODUCTION

Acute myeloid leukemia (AML) is a malignant clonal hematological disease characterized by the expansion of primitive immature cells and the destruction of normal hematopoietic function. Its long-term prognosis has not improved significantly over the past few decades, and its 5-year overall survival (OS) rate is 30% for patients over 20 years of age and less than 10% for patients over 65 years of age.^1^ Immunotherapy has shown promising results in a variety of cancers, but its application in AML still faces great challenges.^2–5^ One reason is that leukemic cells can use autonomous or nonautonomous mechanisms to establish an immunosuppressive bone marrow microenvironment (BMM) to escape the immune killing directed by CD8^+^ T cells.^6^ Therefore, the components of BMM related to immune escape must still be explored for developing new precision immunotherapy strategies.

Tissue-resident memory (TRM) T cells are noncirculating memory T cells that can persist in tissues and contribute to the life-long protection of regional tissues.^7–10^ These cells were first found in barrier organs, such as skin, lungs, intestines, and female reproductive tract.^11^ In recent decades, it has been found in virtually every tissue, including liver, kidney, brain, spleen, lymph node, thymus, and bone marrow (BM).^8–10^ TRM cells typically express tissue-resident markers CD69 and CD103 and lack export-related molecules (eg, CD62L, CCR7, and S1PR1).^7^ Marked by their CD69 expression, the CD4^+^ and CD8^+^ memory T cells in the BM of human and mice have been discovered sharing a similar gene expression profile to the TRM cells in other tissues.^11,12^ Moreover, the pool of BM TRM cells is expandable upon peripheral or systemic antigenic rechallenge.^12,13^ Over the past few years, TRM cells have been found to infiltrate a wide range of solid tumors, and the accumulation of TRM cell-related phenotypic and genetic signatures has been associated with patient prognosis and immunotherapy response.^14^ However, no study has explored the changes of TRM cells in the BM of patients with AML. In the present work, we report for the first time the changed distribution of CD8^+^ TRM-like T cells (CD69^+^ CD45RA^−^ CCR7^−^) in the BM of patients newly diagnosed with AML (ND-AML) and their relevance to the OS. The findings might be critical to the understanding of BMM complexity and contribute to the exploration of new immunotherapies targeting BM TRM-like cells in AML.

## 2. MATERIALS AND METHODS

### 2.1. Sample collection and preparation

In this study, 57 peripheral blood (PB) samples and 49 BM samples were obtained from patients with ND-AML; of which, 41 were derived from the same donor (PB, median age and range in years: 58, 11–88; BM, median age and range in years: 61, 14–84). Unpaired blood samples were collected from 43 healthy individuals (HI, median age and range in years: 54, 13–85), and unpaired BM samples were collected from 10 hematopoietic stem cell (HSC) transplantation donors. In addition, 6 patients with iron deficiency anemia and 2 patients who underwent joint replacement surgery (median age, range: 49.5, 17–76) served as the control group. All the patients were diagnosed at the Institute of Hematology, First Affiliated Hospital of Jinan University (Guangzhou, Guangdong, China) between 2017 and 2022. The sample details are listed in Table 1. All samples were collected with informed consent, and ethical approval was solicited from the Institutional Review Board of Jinan University (No. JNUKY-2022-089). All procedures were conducted following the guidelines of the Medical Ethics Committees of the Health Bureau of the Guangdong Province in China. All the samples were freshly obtained and subjected to immediate preparation.

**Table 1:** Clinical information summary of samples from ND-AML and HI.

	ND-AML		HI	
	PB (n = 57)	BM (n = 49)	PB (n = 43)	BM (n = 18)
Age (y, median, range)	58 (11–88)	61 (14–84)	54 (13–85)	49.5 (17–76)
Male/female	26/31	25/24	25/18	10/8
Number of paired samples	41		0	
WBC (×10^9^ L) (median, range)	13.545 (0.87–318.41)	\	\	\
Blasts % (median, range)	\	64.25 (20–92.5)	\	\

AML = acute myeloid leukemia, BM = bone marrow, HI = healthy individual, ND-AML = newly diagnosed AML, PB = peripheral blood, WBC = white blood cell.

### 2.2. Flow cytometry

Cell surface staining for flow cytometry was performed using the following antibodies: CD45-BU395 (clone HI30; BD, San Jose, California), CD3-AF700 (clone UCHT1, BD), CD8-APC-H7 (clone SK1, BD), CD4-APC-H7 (clone RPA-T4, BD), CCR7-BV605 (clone 3D12, BD), CD45RA-BV510 (clone HI100, BD), CD69-PerCP-Cy5.5 (clone FN50, BD), CD69-PE-Cy7 (clone FN50, BD), PD1-BV421 (clone MIH4, BD), TIGIT-PE (clone A15153G; BioLegend, San Diego, California), CD226-PE-Cy7 (clone 11A8; BioLegend), CD27-PE (clone O323; BioLegend), CD28-PerCP-Cy5.5 (clone CD28.2; BioLegend), and CD57-APC (clone NK-1; BD). These antibodies were used to analyze surface receptors in 2 different panels. Isotype-matched antibodies labeled with proper fluorochromes were used as negative controls. Extracellular staining was performed in accordance with the manufacturer’s instructions. The CCR7-BV605 fluorescent antibody must be stained independently and was first stained at 37°C for 10 minutes. For intracellular staining, the sample was first prepared as a single-cell suspension. After the cell surface was stained, the cells were fixed and permeabilized using the BD Pharmingen™ Transcription Factor Buffer Set and stained with intracellular antibodies for 40 to 50 minutes at 4°C. The intracellular antibodies used were Eomes-FITC (clone WD1928; Invitrogen, Carlsbad, California), TOX-eFluor 660 (clone TXRX10; Invitrogen), T-bet-PerCP-Cy5.5 (BioLegend, clone 4B10), and Ki-67-PE-CF594 (BioLegend, clone Ki-67). Volume 20 μL of absolute count microsphere (Thermo, Waltham, Massachusetts) was added to the samples for absolute number analysis. The cells were examined using a BD Fortessa flow cytometer (BD Biosciences, San Jose, California), and data were analyzed with Flowjo 10.8.1 software.

### 2.3. Analysis of single-cell proteogenomic dataset

The single-cell proteogenomic dataset includes the expression levels of 97 surface markers and 462 mRNAs across 49,057 cells obtained from 3 young, 3 aged, and 3 AML human BM samples. These data were obtained from figshare database (https://figshare.com/). Further detailed information about this dataset can be found in the article authored by Triana et al.^15^ The single-cell sequencing datasets were reanalyzed using the Seurat package (cite Seurat paper). RNA UMI counts were log-normalized, and antibody UMI counts were centered using log ratio normalization by Seurat “NormalizedData” function. The normalized matrices were concatenated and integrated across the samples by Seurat integration method, and the identified “anchors” between dataset pairs were used to eliminate the biological and technical batch effects of the samples. Scaling was then performed with “ScaleData” function. “RunPCA” function was adopted for dimension reduction analysis, and “ElbowPlot” function was utilized to select suitable dimensionality. Different resolution parameters for unsupervised clustering were tested to find the best numbers of clusters. Nonlinear dimensional reduction was performed by “RunUMAP” function.

The T cells were identified through unsupervised clustering analysis by utilizing the expression levels of CD3 genes and surface markers and then further classified into 14 distinct cell types according to the average gene expression profiles of well-known marker genes. Three naive T-cell clusters (CD4^+^ naive, CD8^+^ naive, and naive γδ T) were characterized by high CCR7 and SELL expression. γδ T cells were defined by high T cell receptor delta constant (TCRD) expression. Two clusters of effector T cells (CD4^+^ TEFF and CD8^+^ TEFF) exhibited a high expression of effector cytokines (NKG7, GZMA, and GNLY). Two central memory T cell clusters (CD4^+^ TCM and CD8^+^ TCM) and 2 effector memory T cell clusters (CD4^+^ TEM and CD8^+^ TEM) exhibited a low expression of naive and effector cytokines. The TEM cells displayed lower expression of CCR7 and SELL than the TCM cells. CD8^+^ CD103^+^ TRM cells were defined by their ITGAE expression (CD103). Finally, the distinct clusters of CD4^+^ CD69^+^ and CD8^+^ CD69^+^ T cells were identified from their CD69 expression. On the basis of NKG7, GZMA, and GNLY expression, the enrichment scores of cytotoxicity were calculated using the “AddModuleScore” function in Seurat at the single-cell level.

### 2.4. Survival analysis

This research was a retrospective case–control study. Forty-two AML cases with available data were included in the survival analysis. The patients (ages 14–80 years) registered at the First Affiliated Hospital of JNU between 2017 and 2022 with an initial diagnosis of AML, and no restrictions on sex or ethnicity were set for their inclusion. The patients diagnosed with M3 subtype were excluded from the survival analysis. Detailed diagnosis including age, sex, FAB typing, gene mutation, and karyotypes and treatment information are shown in Table S1, http://links.lww.com/BS/A97. OS time was defined as the time from a new diagnosis to the survival time or death from any cause. X-tile was used to categorize the patients into CD8^+^ TRM-like high and low groups, CD69^−^ TEM high and low groups, CD69^+^ T cell high and low groups, and groups under and above 60 years old for survival comparison (Table S2, http://links.lww.com/BS/A97). Survival difference was analyzed using the Kaplan–Meier method and Cox regression analysis, and the prognostic effect of the mean expression of *CD8A*, *CD69*, and *TOX* genes in the AML BM sample dataset was evaluated using the Kaplan–Meier plotter (Kaplan–Meier plotter [AML] [kmplot.com]). The AML BM sample datasets were obtained from the Gene Expression Comprehensive database (GSE1159, GSE12417, GSE37642, GSE6891, and GSE8970) with an OS of 60 months and a total sample size of 787. The parameter selection in the Kaplan–Meier plotter is shown in Table S3, http://links.lww.com/BS/A97.

### 2.5. Statistical analysis

All data are represented as medians plus 95% confidence intervals. Statistical analyses were performed with Prism 9.5.0 (GraphPad Software, Inc.) and R (version 4.1.0) using Mann–Whitney *U* test or Wilcoxon test and Fisher exact test depending on the experimental design. The Kaplan–Meier method and univariate and multivariate Cox regression analysis were used to analyze between-group survival differences.

## 3. RESULTS

### 3.1. High percentage of CD8^+^ TRM-like cells in the BM of patients with AML

**Figure 1.**
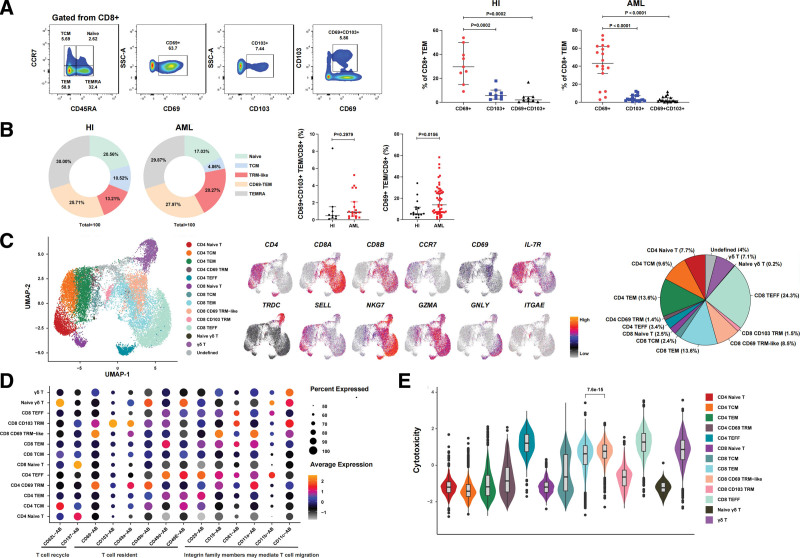
CD8^+^ TRM-like cells increased in the bone marrow of newly diagnosed AML patients. (A) Flow cytometry gating plots show that CD8^+^ T cells are first separated into differentiation stage by CD45RA and CCR7 (naive [CCR7^+^CD45RA^+^], TCM [CCR7^+^CD45RA^−^], TEM [CCR7^−^CD45RA^−^], and TEMRA [CCR7^−^CD45RA^+^]). The CD69^+^, CD103^+^, and CD69^+^CD103^+^populations in TEM were further analyzed. The scatter plot shows that the CD8^+^ TEM cell subpopulation in the patients with ND-AML and HI significantly expressed CD69 but not CD103. The number (n) of samples included in each subset analysis was as follows: HI (n = 9); AML (n = 18). (B) Proportion of naive, TCM, TRM-like, CD69^−^ TEM, and TEMRA cells in the BM CD8^+^ T cell subsets of patients with ND-AML and HIs. The scatter plot shows that compared with that of HIs, the BM of patients with ND-AML contains a higher proportion of CD69^+^ CD8^+^ TEM cells. The number (n) of samples included in each subset analysis was as follows: HI (n [CD69^+^CD103^+^ TEM] = 9, n [CD69^+^ TEM] = 15), AML (n [CD69^+^CD103^+^ TEM] = 18, n [CD69^+^ TEM] = 49). (C) Left: UMAP visualization of BM T cell single cell clusters for single-cell proteogenomic dataset. Different cell types were depicted with distinct colors. Middle: Projection of marker genes expression levels for BM T cells. Right: Proportion of different T cell subsets in BM. (D) Bubble plot of selected functional antibody AB for each cell cluster of T cells. Color scale indicates the mean of normalized expression of marker genes in each cell type, and dot size is proportional to the percentage of cells within each cell cluster expressing antibody. (E) Violin plots with boxplot insert of cytotoxic functional scores for each subtype of T cells. *P* values were calculated by Wilcoxon rank sum test (2-sided). Unpaired *t* test and Mann–Whitney *U* test were used for unpaired sample analysis. DN = newly diagnosed, HI = healthy individual, ns = not significant, TEM = effector memory T cell, TEMRA = effector memory T cell re-expressing CD45RA, TCM = central memory T cell, TRM-like = tissue-resident-like T cell, UMAP = uniform manifold approximation and projection.

Given that CD69 is predominantly expressed by BM memory T cells but hardly expressed by blood memory T cells,^16^ we analyzed its expression in the T cells from the PB and BM of HIs and patients with AML. The results showed that the CD8^+^CD69^+^ T cells were abundant in the BM (HI: *P* = .0001, AML: *P* = .0001) but not in the PB; however, no significant difference in CD4^+^CD69^+^ T cells was observed between the PB and BM (HI: *P* = .6409, AML: *P* = .2481) (Figure S1A, http://links.lww.com/BS/A97). Approximately 60% CD8^+^CD69^+^ T cells were distributed in the TEM (CCR7^−^CD45RA^−^) subpopulation (Figure S1B, http://links.lww.com/BS/A97). These results were consistent with previous findings in HI^17^ and further demonstrated that CD8^+^CD69^+^ TEM cells also exist in the BM of patients with AML. Furthermore, we compared the single and double expression frequency of CD69 and CD103 on BM CD8^+^ TEM cells and found that regardless of the HI or AML cohort, the CD8^+^ TEM subset predominantly expressed CD69 (HI average: 31.46%, AML average: 42.45%). Only a few of these cells expressed CD103 (HI, 6.88%; AML, 4.26%) alone or in combination with CD69 (HI average: 3.93%, AML average: 3.06%) (**Fig. 1A**). Thus, we called the CD8^+^ TEM cells expressing CD69 alone as CD8^+^ TRM-like cells to discriminate them from the bona fide TRM cells expressing CD69 and CD103 in most of the other tissues.

Comparison of TRM and TRM-like cells between HI and patients with AML revealed that the proportion of TRM-like cells significantly increased (HI vs AML, 9.85 ± 8.89 vs 18.24 ± 14.08, *P* = .0156) in the AML cohort. No significant difference was observed in the TRM subpopulation (*P* = .2979, **Fig. 1B**). Using the single-cell proteogenomic reference maps of the hematopoietic system of human BM,^15^ we reanalyzed the CD3 T cell population in the HI and patients with AML. We confirmed that more CD69^+^ TRM-like CD8^+^ T cells accumulated in the human BM than CD103^+^ TRM cells and only a few CD4^+^ TEM cells expressed CD69 in the human BM (**Fig. 1C** and Figure S2, http://links.lww.com/BS/A97). The bubble chart in Figure 1D further confirms the tissue-resident characters of the CD8^+^ TRM-like population in the BM. Except for CD69, the CD8^+^ TRM-like cells showed a higher expression of tissue-resident makers CD49a and CD11a/CD18 (LFA1)^18,19^ compared with other T cell populations. The CD8^+^CD103^+^ TRM and CD8^+^CD69^+^ TRM-like subsets in the BM also highly expressed CD11c, which is abundantly expressed in monocytes and macrophages.^20,21^ Using the genomic data, we further evaluated the cytotoxic capacity of each T cell subset and found that the CD4^+^ and CD8^+^ TEFF subsets had the strongest cytotoxicity, followed by γδ T cells and CD8^+^ TRM-like and CD8^+^ TEM subsets. In particular, the CD8^+^ TRM-like subset had a higher cytotoxic score than the CD8^+^CD103^+^ TRM population and even higher than the CD8^+^ TEM subset (*P* = 7.6 × 10^−15^) (**Fig. 1E**).

The above results jointly proved that the abundant BM CD8^+^ TEM cells predominantly express CD69 rather than CD103 in the HIs and patients with AML. These CD8^+^CD69^+^ TEM cells adopt tissue-resident characteristics. In addition, the CD8^+^ TRM-like population has expanded in the AML cohort.

### 3.2. High proportion of BM CD8^+^ TRM-like subset associated with poor prognosis in patients with ND-AML

**Figure 2.**
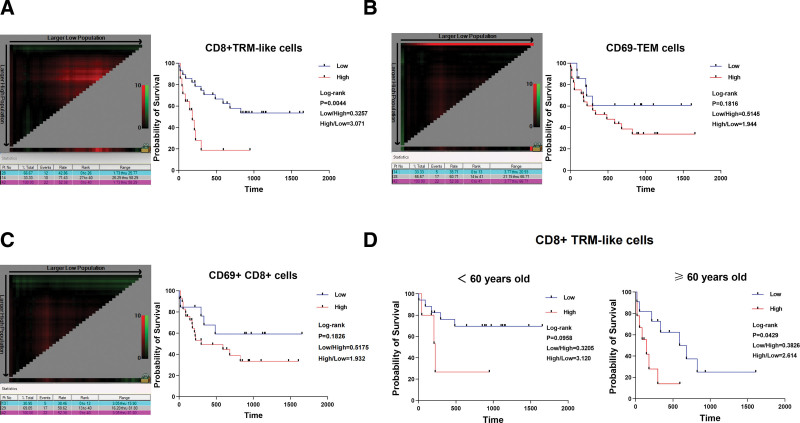
High proportion of CD8^+^ TRM-like subpopulation in the bone marrow is associated with poor prognosis. (A) The figure on the left shows the truncation value of the CD8^+^ TRM-like subset using X-tile. OS analysis of high and low proportions of CD8^+^ TRM-like subpopulations in the BM of patients with ND-AML. A high proportion of subpopulations was significantly associated with poor prognosis. The number (n) of samples included in each subset was as follows: n = 42, low (n = 28, range: 1.73–25.77) and high (n = 14, range: 26.25–58.29). (B) The figure on the left shows the truncation value of the CD69^−^ TEM subset using X-tile. OS analysis of high and low proportions of CD69^−^ TEM subpopulations in the BM of patients with ND-AML. The high or low proportion of subpopulations was shown to be independent of patient survival. The number (n) of samples included in each subset was as follows: n = 42, low (n = 14, range: 3.77–20.93) and high (n = 28, range: 21.15–66.71). (C) The figure on the left shows the truncation value of the CD69^+^CD8^+^ subset using X-tile. OS analysis of high and low proportions of CD69^+^CD8^+^ T cell subpopulations in the BM of patients with ND-AML. The high or low proportion of subpopulations was shown to be independent of patient survival. The number (n) of samples included in each subset analysis was as follows: n = 42, low (n = 13, range: 3.05–15.90) and high (n = 29, range: 16.20–81.80). (D) OS analysis of high and low proportions of CD8^+^ TRM-like subgroups in the BM of patients with ND-AML who are 60 y old or older. The CD8^+^ TRM-like subset was significantly associated with poor prognosis in patients aged 60 y and older. The number (n) of samples included in each subset analysis was as follows: <60 y old (n = 22, low [n = 17], high [n = 5]), ≥60 y old (n = 20, low [n = 11], high [n = 9]). Log-rank (Mantel–Cox) test was used for sample analysis. BM = bone marrow, ND = newly diagnosed, ns = not significant, OS = overall survival, TEM = effector memory T cell, TRM-like = tissue-resident-like T cell.

To further explore whether the presence of CD8^+^ TRM-like subsets is related to AML prognosis, we tracked the survival data of all the patients with ND-AML (excluding the patients with M3 subtype). We used X-tile to divide the proportion of CD8^+^ TRM-like subset into high and low groups and then performed Kaplan–Meier survival analysis to examine the survival rate of patients. The results showed that the high proportion of CD8^+^ TRM-like subset (26.25%–58.29%) in the BM of patients with ND-AML was associated with poor OS (*P* = .0044, **Fig. 2A**). We also analyzed the relationship of CD69^−^ TEM subpopulation and CD69^+^CD8^+^ T cell subpopulation with patient OS rate and found no statistical association (CD69^−^ TEM: *P* = .1816; CD69^+^CD8^+^ T cell: *P* = .1826; **Fig. 2B and C**). Univariate and multivariate Cox regression analysis further found that age and the high proportion of BM CD8^+^ TRM-like subset were independent risk factors that can affect the survival of patients. Owing to the limited sample cohort, the hazard ratio between intermediate/adverse and favorable groups (based on the 2017 version of the European Leukemia Net) was not significantly different (Table 2).

**Table 2:** Univariate and multivariate analysis of the overall survival in the ND-AML patients.

Factors	Univariate analysis		Multivariate analysis	
	HR (95% CI)	*P*	HR (95% CI)	*P*
Age (≥60 vs <60 y)	2.632 (1.094–6.336)	.031	3.269 (0.998–10.701)	.050
Gender (male vs female)	1.106 (0.478–2.561)	.814	0.471 (0.175–1.268)	.136
CD8^+^ TRM-like cells (≥26% vs <26%)	3.355 (1.393–8.081)	.007	3.484 (1.050–11.560)	.041
CD69^−^ TEM (≥21% vs <21%)	1.948 (0.718–5.286)	.191	2.023 (0.691–5.922)	.198
CD69^+^CD8^+^ T cell (≥16% vs <16%)	1.952 (0.715–5.327)	.191	1.080 (0.224–5.202)	.924
Prognosis (intermediate/adverse vs favorable)	2.034 (0.594–6.971)	.259	2.252 (0.551–9.206)	.259

95% CI = 95% confidence interval, AML = acute myeloid leukemia, HR = hazard ratio, ND-AML = newly diagnosed AML.

The above results indicated that the BM CD8^+^ TRM-like subset is quite a sensitive survival prediction index for ND-AML. Thus, we used the Fisher exact test to explore whether patients with high and low BM CD8^+^ TRM-like subset have different clinical characteristics. The findings showed no differences in age, gender, chromosomal variations, genetic mutations, and patient risk classification between the patients with high and low proportions of CD8^+^ TRM-like subset (Table 3). Based on this result, we further used the Kaplan–Meier method to explore the survival of patients with AML under and beyond 60 years of age. The result showed that a high proportion of CD8^+^ TRM-like subpopulation in older patients was significantly associated with poor prognosis (**Fig. 2D**, *P* = .0429), but no significant difference was observed in younger patients (**Fig. 2D**, *P* = .0958). Overall, these finding showed that an increased proportion of BM CD8^+^ TRM-like subset could be used to predict the inferior survival of patients with ND-AML, especially the elderly.

**Table 3:** Correlation between the proportion of CD8^+^ TRM-like subpopulation and clinical characteristics of ND-AML patients.

Variables	CD8^+^ TRM-like cells		
	Low-expression N = 28 (1.73–25.77)	High-expression N = 14 (26.25–58.9)	*P* value
Age segmentation			.191
<60	17 (60.71%)	5 (35.71%)	
≥60	11 (39.29%)	9 (64.29%)	
Gender			.742
Male	15 (53.57%)	9 (64.29%)	
Female	13 (46.43%)	5 (35.71%)	
Chromosome			.707
Abnormal	8 (42.11%)	6 (54.55%)	
Normal	11 (57.89%)	5 (45.45%)	
WBC (×10^9^ L)			.098
BM blast cell (%)			.785
*DNMT3*			.279
Negative	19 (86.36%)	13 (100.00%)	
Positive	3 (13.64%)	0 (0.00%)	
*EVI1*			>.999
Negative	19 (86.36%)	12 (92.31%)	
Positive	3 (13.64%)	1 (7.69%)	
*FLT-3*			.721
Negative	13 (59.09%)	9 (69.23%)	
Positive	9 (40.91%)	4 (30.77%)	
*NPM1*			.2735
Negative	18 (81.82%)	13 (100.00%)	
Positive	4 (18.18%)	0 (0.00%)	
*CEBPA*			.726
Negative	15 (68.18%)	8 (61.54%)	
Positive	7 (27.27%)	5 (38.46%)	
IDH1/2			>.999
Negative	21 (95.45%)	13 (100.00%)	
Positive	1 (4.55%)	0 (0.00%)	
Risk stratification			.081
Favorable	7 (28.00%)	1 (7.14%)	
Intermediate	11 (44.00%)	6 (42.86%)	
Adverse	7 (28.00%)	7 (50.00%)	
Subtype, n (%)			.661
M4&M5	12 (42.86%)	7 (50.00%)	
Excludes M4&M5	16 (57.14%)	7 (50.00%)	

AML = acute myeloid leukemia, BM = bone marrow, ND-AML = newly diagnosed AML, WBC = white blood cell.

**Figure 3.**
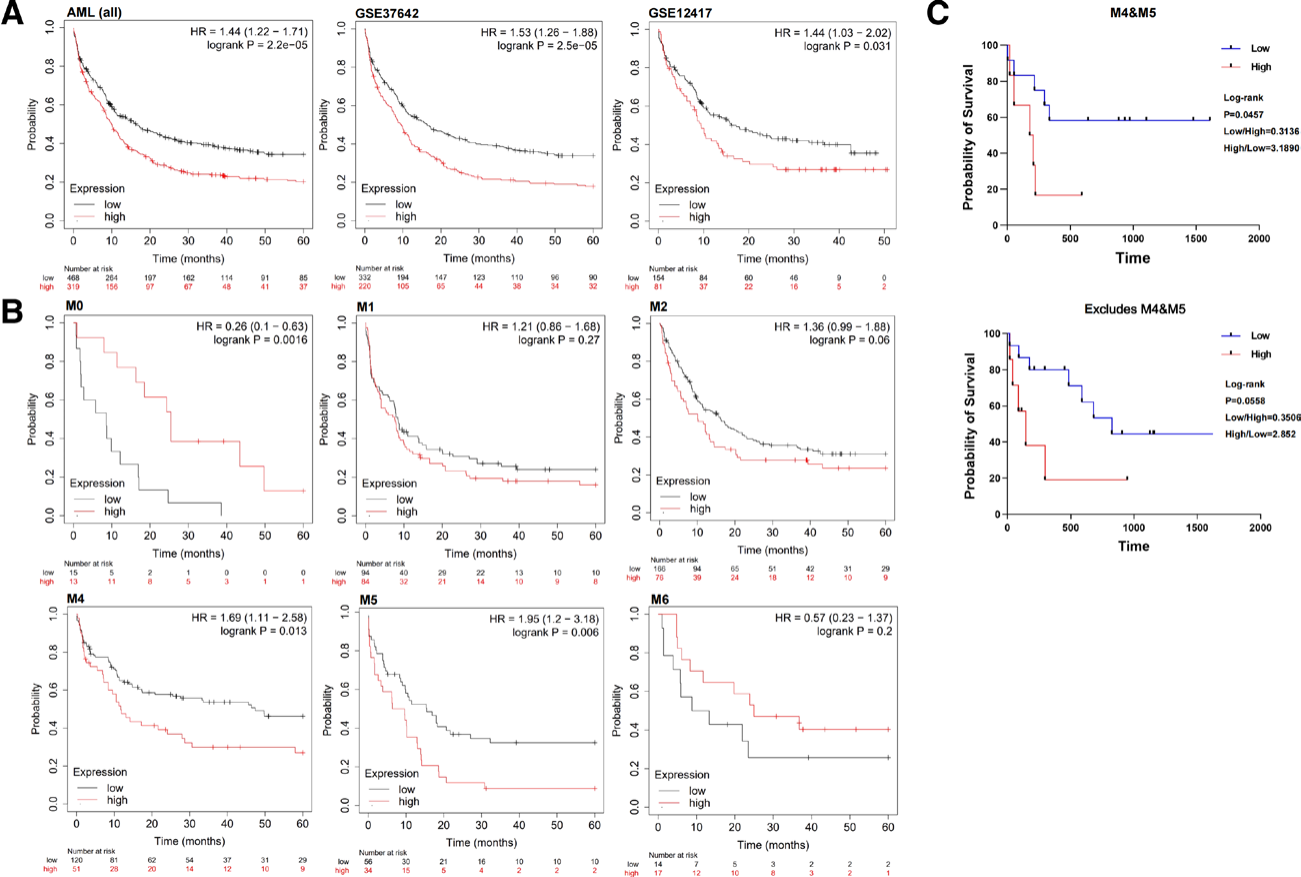
High expression levels of *CD8A*, *CD69*, and *TOX* genes in the bone marrow associated with poor prognosis of patients with AML. (A–B) The AML dataset of “Kaplan–Meier Plotter” was used to analyze the correlation of the expression of *CD8A*, *CD69*, and *TOX* with patient OS. (A) The results of no typing or different databases, (B) the results of M0–M6 typing (excluding M3). (C) The collected ND-AML samples were used to analyze the correlation between the expression of CD8^+^ TRM-like subgroups and patient OS. The number (n) of samples included in each subset analysis was as follows: M4&M5, n = 19; excludes M4&M5, n = 23. Log-rank (Mantel–Cox) test was used for sample analysis. AML = acute myeloid leukemia, HR = hazard ratio, ND = newly diagnosed, ns = not significant, OS = overall survival, TRM-like = tissue-resident-like T cell.

We further confirmed whether the BM CD8^+^ TRM-like population could predict the OS of patients with ND-AML in a validation cohort. Basing on the above result, we selected *CD8A, CD69*, and *TOX* genes as the key characteristic gene of the CD8^+^ TRM-like population. Using the AML dataset (GSE1159, GSE12417, GSE37642, GSE6891, and GSE8970) included in “Kaplan–Meier plotter” (Kaplan–Meier plotter [AML] [kmplot.com]), we found that the high expression of *CD8A*, *CD69*, and *TOX* genes predicted a poor 5-year OS in 787 patients with AML in these 5 datasets. Two independent datasets (GSE12417 and GSE37642) comprising 235 and 552 AML cases, respectively, also yielded the same results (**Fig. 3A**). Survival analysis by FAB subtypes revealed that the high expression of *CD8A*, *CD69*, and *TOX* genes could predict inferior OS in patients with M4 and M5 subtypes (**Fig. 3B**). The above analysis was conducted using the bulk mRNA data from the BM of patients with AML, which might be an inaccurate representation of the actual amount of TRM-like cells in the sample. Using single-cell RNA sequence data or flow cytometry data to confirm this result is necessary. Within our cohort, the result showed that the high percentage of BM CD8^+^ TRM-like subset also predicted poor prognosis in the M4 + M5 samples (*P* = .0457). When the M4 and M5 subtypes were excluded, the *P* value loss of significance in the remaining samples (*P* = .0558) (**Fig. 3C**). Therefore, the BM CD8^+^ TRM-like population is closely associated with the survival of patients with ND-AML and could be a valuable immunological marker for predicting the survival of patients with AML, especially in the elderly and those with M4/M5 subtypes.

### 3.3. BM CD8^+^ TRM-like subset highly expresses co-inhibitory molecules and stays in a quiescent state

**Figure 4.**
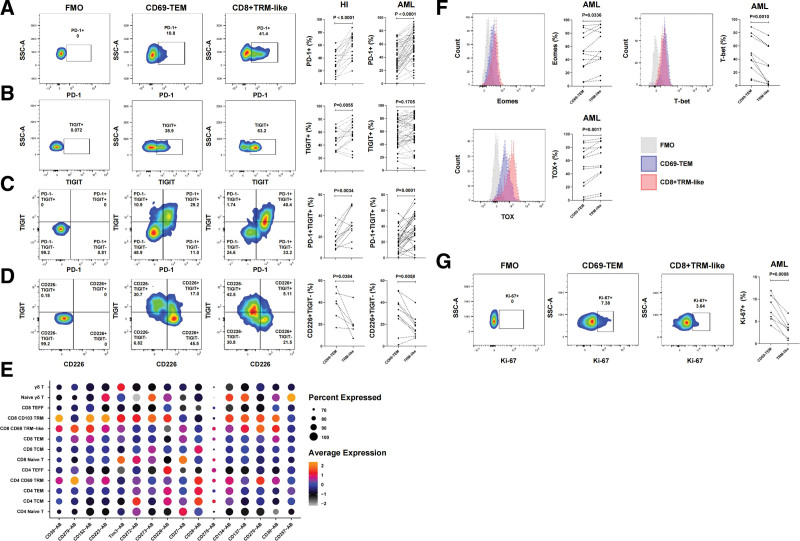
Bone marrow CD8^+^ TRM-like subset shows T cell exhaustion phenotype and stay in quiescent state. (A–D) Flow cytometry was used to analyze the expression of PD-1 (A), TIGIT (B), PD-1^+^TIGIT^+^ (C), and TIGIT^−^CD226^+^ (D) in the CD69^−^ TEM and CD8^+^ TRM-like cell subsets in the BM of HIs and patients with ND-AML. Compared with CD69^−^ TEM, the CD8^+^ TRM-like subset highly expressed PD-1 and TIGIT in the BM of HIs and patients with AML. The number (n) of samples included in each subset was as follows: HI (n [PD-1^+^] = 18, n [TIGIT^+^] = 18, n [PD-1^+^TIGIT^+^] = 11, n [CD226^+^TIGIT^−^] = 6), AML (n [PD-1^+^] =48, n [TIGIT^+^] = 49, n [PD-1^+^TIGIT^+^] = 30, n [CD226^+^TIGIT^−^] = 10). (E) Bubble plot of selected functional antibody AB for each cell cluster of T cells. Color scale indicates the mean of normalized expression of marker genes in each cell type, and dot size is proportional to the percentage of cells within each cell cluster expressing antibody. (F–G) Flow cytometry was used to detect the distribution and frequency of Eomes, T-bet, TOX (F), and Ki-67 (G) on the CD69^−^ TEM and CD8^+^ TRM-like cell subsets in the BM of patients with ND-AML. Compared with CD69^−^ TEM, the CD8^+^ TRM-like subset expressed Eomes and TOX and lowly expressed T-bet. The CD8^+^ TRM-like subset lowly expressed Ki-67. The number (n) of samples included in each analysis was as follows: Eomes, n = 12, T-bet, n = 11, TOX, n = 12, Ki-67, n = 8. Paired *t* test was used for paired sample analysis. AML = acute myeloid leukemia, BM = bone marrow, HI = healthy individual, ND = newly diagnosed, ns = not significant, PD-1 = programmed death receptor-1, TEM = effector memory T cell, TIGIT = T cell immunoreceptor with Ig and ITIM domains, TRM-like = tissue-resident-like T cell.

To further analyze why a high CD8^+^ TRM-like proportion is associated with poor prognosis in AML, we tested the immune checkpoints (ICPs) and PD-1 and TIGIT expression in CD8^+^ TRM-like cells. The CD69^−^ TEM subset at the same differentiation stage was used as a control. The results showed that the CD8^+^ TRM-like subset from the HI (PD-1^+^, *P* < .0001; TIGIT^+^, *P* = .0055; PD-1^+^TIGIT^+^, *P* = .0034) and AML (PD-1^+^, *P* < .0001; TIGIT^+^, *P* = .1705; PD-1^+^TIGIT^+^, *P* = .0001) samples had a higher proportion of PD-1- and TIGIT-positive cells (single and dual positive) than its CD69^−^ TEM comparable subset (**Fig. 4A–C**). CD226 is a costimulatory molecule that can bind with CD155 or LFA1 to mediate T cell activation. TIGIT can compete with CD226 in binding to CD155 with high affinity, thereby limiting CD226-mediated activation. We also compared CD226 expression between the CD8^+^ TRM-like and CD69^−^ TEM subsets and found that the former had a significantly lower proportion of TIGIT^−^CD226^+^ than the latter in the HI and patients with AML (HI, *P* = .0384; AML, *P* = .0058) (**Fig. 4D**). Based on the single-cell proteogenomic data, we further confirmed that the CD8^+^ TRM-like subset highly expressed PD-1 (CD279) compared with the other CD8^+^ cell subpopulations and showed the lowest CD266 expression among all the annotated T cell subsets (**Fig. 4E**). In addition, the CD8^+^ TRM-like subset had a higher expression of ICPs such as CD39, CD152 (CTLA-4), CD223 (LAG-3), and TIM-3 compared with the CD69^−^ TEM subset (**Fig. 4E**). These results indicated that the BM CD8^+^ TRM-like population may stay highly exhausted. Thus, we further analyzed the transcription factors that are closely correlated with the function of T cells, including Eomes, T-bet, and Tox. Figure 4F shows that in the BM of patients with AML, the expression frequency of Eomes and Tox in the CD8^+^ TRM-like subset was also higher than that in the CD69^−^ TEM subpopulation (Eomes: *P* = .0336; TOX, *P* = .0017) and the expression frequency of T-bet was relatively reduced (T-bet, *P* = .0010).

**Figure 5.**
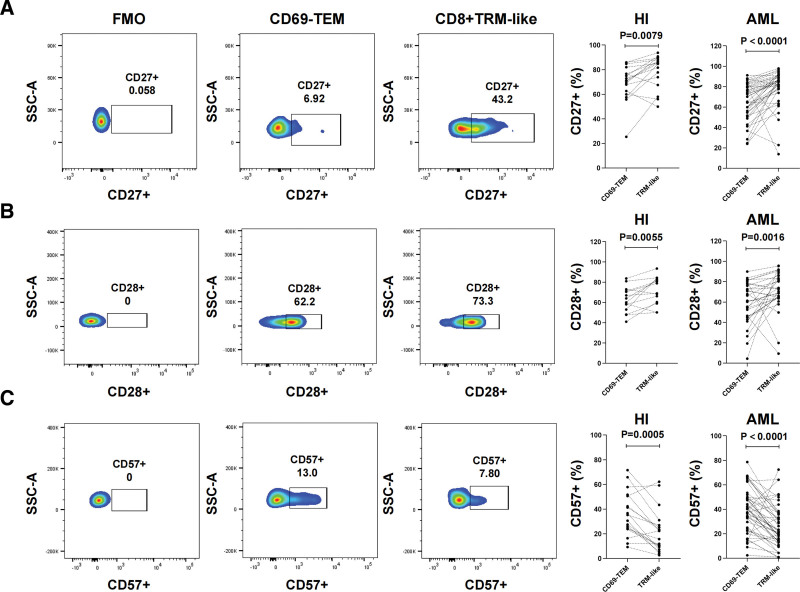
Frequency of CD27, CD28, and CD57 expression on the bone marrow CD8^+^ TRM-like subset of HIs and patients with ND-AML. (A–C) Flow cytometry was used to detect the distribution and frequency of CD27 (A), CD28 (B), and CD57 (C) on the CD69^−^ TEM and CD8^+^ TRM-like cell subsets in the BM of HIs and patients with ND-AML. Compared with CD69^−^ TEM, the CD8^+^ TRM-like subset expressed CD27 and CD28 and low expression of CD57. The number (n) of samples included in each subset analysis is shown as follows, HI (n [CD27^+^] = 17, n [CD28^+^] = 14, n [CD57^+^] = 18), AML (n [CD27^+^] = 41, n [CD28^+^] = 32, n [CD57^+^] = 47). Wilcoxon test and paired *t* test were used for paired sample analysis. AML = acute myeloid leukemia, HI = healthy individual, ND = newly diagnosed, ns = not significant, TEM = effector memory T cell, TRM-like = tissue-resident-like T cell.

The above results showed that BM CD8^+^ TRM-like cells represent a dysregulated T cell population expressing several ICPs. Proliferative capacity is an important component of T cell fitness, and Ki-67 protein is a well-validated marker of cell proliferation. Here, we found that Ki-67 expression was significantly lower in the CD8^+^ TRM-like subset than in the CD69^−^ TEM subset (*P* = .0008, **Fig. 4G**). This result hinted that BM CD8^+^ TRM-like cells stay in a quiescent state. T cell senescence is also related to tumor immune evasion and poor prognosis.^22,23^ As T cells differentiate, replicate, and age, their expression of costimulatory molecules CD27 and CD28 decreases and that of CD57 increases.^24^ We further compared the expression levels of these molecules in the 2 subpopulations. Different from the expected results, the CD8^+^ TRM-like cells in the BM had higher CD27 (HI, *P* = .0079; AML, *P* < .0001) and CD28 (HI, *P* = .0055; AML, *P* = .0016) expression and lower CD57 expression (HI, *P* = .0005; AML, *P* < .0001) compared with the CD69^−^ TEM cells (**Fig. 5A–C**). On this basis, we further analyzed the expression of costimulatory molecules in each T cell subpopulation in the above single cell-protein-genome data. We found that CD27, CD30, CD137, and other costimulatory molecules were highly expressed, suggesting that the CD8^+^ TRM-like subpopulation may still have proliferation potential (**Fig. 4E**).

## 4. DISCUSSION

For the first time, our study identified an increased proportion of CD8^+^ TRM-like cells in the BM of patients with ND-AML and found its association with poor OS. TRM cells can infiltrate a variety of solid tumors,^14^ and the increased proportion of CD8^+^ TRM-like cells in several tumor tissues has been associated with improved responses to immunotherapy and favorable clinical outcomes.^25^ For example, in human lung and hepatocellular carcinoma, the increased density of PD-1^+^CD103^+^CD8^+^ TRM cells in immunotherapy-naive tumors is associated with improved outcomes after anti-PD(L)1 therapy.^9,26^ These positive correlation examples seem to have a similar mechanism; even though TRM cells highly express several ICPs and display exhaustion phenotypes, they have relatively high cytotoxic capacity and could limit tumor progression.^27^ In the present study, the BM CD8^+^ TRM-like subset also exhibited the characteristics of T cell exhaustion, such as high percentages of PD-1, TIGIT, and TOX. However, the opposite survival correlation was observed. Similar results were found in the study of Zhou et al^28^ on prostate cancer, with the high proportion of tumor-infiltrating CD103^+^ cells correlated with reduced biochemical recurrence-free survival. The above positive and negative correlations between the accumulation of TRM cells in the TME and the prognosis of patients hinted that different tumor may have varying responses to the TRM-accumulated TME. A recent work reported that tumor-specific or bystander TRM-like cells present before tumor onset can shape the evolution of tumor immunogenicity and boost immune cell recruitment, causing tumor immune evasion through the loss of MHC class I protein expression and resistance to immune checkpoint inhibitors (ICIs).^29^ BM is a TRM-like cell abounded field; whether the poor prognosis of patients with a high CD8^+^ TRM-like proportion may be attributed to the loss of MHC class I caused by high immune pressure is worthy of further investigation. Another study found that the accumulation of atypical tissue-resident CD69^+^ terminal effector cells (TEFs, CD8^+^CD57^+^CD45RA^+^) in the BM of patients with myeloma could prevent the differentiation and expansion of clonal myeloma-specific CD8^+^ TEFs and ultimately contribute to myeloma immune escape.^30,31^ They also reported that these CD69^+^ TEF populations produce large amounts of inflammatory cytokines interferon-γ (IFN-γ) and tumor necrosis factor-α (TNF-α) and exhibit low perforin and granzyme expression. Thus, the lack of cytotoxic effects and the enhanced inflammatory function may be another reason for the CD8^+^ TRM-like cells leading to a poor prognosis in AML. Several works recently emphasized that the inflammation cytokine INF-γ in the BM can impact the prognosis of AML.^32^ One study suggested that CD8 T and NK cell IFN-γ production in the BM shapes a unique immunosuppressive microenvironment through HLA-E and CD74 up regulation on leukemic cells from patients with AML exhibiting monocytic differentiation and del7/7q.^32^ Further research is needed to determine whether INF-γ is secreted by the CD8^+^ TRM-like cells in the BM of patients with M4 and M5 phenotypes and whether INF-γ signaling can mediate immune evasion and leukemia blast growth.^32^

We discussed the possible reasons for CD8^+^ TRM-like cell accumulation in the BM that induces leukemia immune escape in AML. Whether this special T cell population can be targeted to develop immune therapy for AML is an interesting question. In our study, we found that the CD8^+^ TRM-like cells in the BM have a high expression of costimulatory molecules CD27 and CD28 and a low expression of Ki-67 (a proliferation marker) and CD57 (a marker of replicative senescence and terminal differentiation. Single-cell proteogenomic analysis also revealed that the CD8^+^ TRM-like subset in the BM highly expresses several other costimulatory molecules such CD278 and CD137. These results indicated that BM CD8^+^ TRM-like cells may remain in a quiescent state without serious replication but maintains a potential to differentiate and give rise to a huge population of T cell progeny. Kumar et al^33^ also reported that human lung and spleen CD8^+^CD69^+^ memory T cells undergo reduced proliferation turnover, which is indicated by low Ki-67 and CD57 expression. Gebhardt et al^34^ proposed that true TRM cells still retain a certain degree of stemness, allowing them to maintain self-proliferation and differentiate into circulating T cells upon encountering antigens. We and other researchers showed that TRM cells have superior proliferation potential even when they remain in a quiescent state. The high ICPs expression may be the reason for their inhibited activation. Thus, using ICIs to activate the antitumor potential of tumor-infiltrating TRM-like cells is a reasonable method for tumor treatment. Several studies reported that TRM or TRM-like cells could directly respond to ICIs by producing effector-like cytolytic exhausted T cells, exhausted T cells in transient or intermediate differentiation states, and progeny downstream cells of terminally exhausted T cells, thereby directly attacking cancer cells to further promote tumor control.^34^ This effect may be attributed to the high expression of costimulatory molecules in TRM cells. As reported by Kim et al,^35^ the expression of costimulatory receptors in CD8^+^ T cells is an important factor for their responsiveness to PD-1 blocking. Qureshi et al^36^ also stated that the increase in CD8 TEM cell proliferation caused by blocking CTLA-4 or PD-1 can be reversed using an anti-CD28 domain antibody. Thus, the TRM-like cells infiltrating tumor tissues may be the real target cell in ICI treatment. For AML and other cancers without significant response to ICI treatment, the successful application of ICIs relies on the discovery of a second immune suppressive brake that functions independently from ICRs. Koyama-Nasu et al^37^ found that CD69 deficiency resulted in a decreased expression of the transcription factor TOX in the tumor-specific CD8^+^ T cells of tumor-draining lymph nodes, promoting the differentiation of stem-like CD8^+^ T cells into functional terminally differentiated CD8^+^ T cells with enhanced antitumor function, and synergizing with anti-PD-1 treatment to render immune refractory B16 melanoma susceptible to immunotherapy. The above results indicated that CD69 acts as an important negative regulator for the differentiation of tumor-specific CD8^+^ T cells. Therefore, targeting CD69 and PD-1 may be a promising immunotherapy for AML.

Overall, we found that the proportion of BM CD8^+^ TRM-like T cell subset is significantly increased in patients with ND-AML and can predict the OS of these patients, especially in the elderly over 60 years old and in patients with M4 and M5 phenotypes. Although this group of cells exhibits the characteristics of exhausted T cells, they have a high proliferative potential. Therefore, reversing the function of this subset using ICIs and another immune-related “brakes” such as CD69 may benefit the survival of some patients with AML.

## ACKNOWLEDGMENTS

This study was supported by grants from the National Natural Science Foundation of China (No. 82000108), the Guangdong Basic and Applied Basic Research Foundation (No. 2020A1515110310), the Guangdong Natural Science Foundation of China (No. 2023A1515010170), NSFC Incubation Project of Guangdong Provincial People’s Hospital (No. KY0120220026), Guangdong Provincial Outstanding Young Medical Talents Supporting Research Foundation (No. KJ012019459), and Key Laboratory for Regenerative Medicine of Ministry of Education Project (No. ZSYXM202001), the National Innovation and Entrepreneurship Training Program for Undergraduate (No. CX23386).

We would like to thank patients for participating in this study. In addition, we would like to thank the Institute of Aging and Regenerative Medicine of Jinan University for providing us with flow cytometry and technical operation.

## ETHICAL APPROVAL

The studies involving human participants were reviewed and approved by Ethics Committee of Institutional Review Board of Jinan University (No. JNUKY-2022-089). The patients/participants provided their written informed consent to participate in this study.

## AUTHOR CONTRIBUTIONS

L.C. and Y.G. collected and analyzed the data. Single-cell proteogenomic data set was analyzed by W.L., O.J.L., D.Y., L.L., X.Y. performed flow cytometry. L.L., C.W., J.L., Z.Y., X.Z., C.L. collected samples and patient information. X.W., X.Y., and S.C. helped to organize the lab experiments. L.X., X.H., and P.Q. conceived the study, designed the experiment, and oversaw the research project. Y.L. helped to revise the manuscript. All authors contributed to this article and have approved the submitted version.

## Supplementary Material
