# Supplementary material for: High percentage of bone marrow CD8+ tissue-resident-like memory T cells predicts inferior survival in patients with acute myeloid leukemia
Source: Blood Sci. 2024 Jun 7;6(3):e00194. doi: 10.1097/BS9.0000000000000194 (PMC11161300; doi:10.1097/BS9.0000000000000194)
Supplement: Supplementary file 1 [file bs9-6-e00194-s001.pdf]

Supplemental Material

List of Content

1. Supplemental Figures

- **Figure S1.** CD69 is mainly expressed by BM memory T cells and mainly distributed in the TEM subpopulation.
- **Figure S2.** T cell subpopulation in single-cell proteogenomic dataset.

2. Supplemental Tables

- **Table S1.** Clinical information of ND-AML
- **Table S2.** The specific cut-off of $CD8^+$ TRM-like, $CD69^-$ TEM, and $CD69^+CD8^+$ T cells.
- **Table S3.** Choice of Kaplan–Meier plotter

1. Supplemental Figures

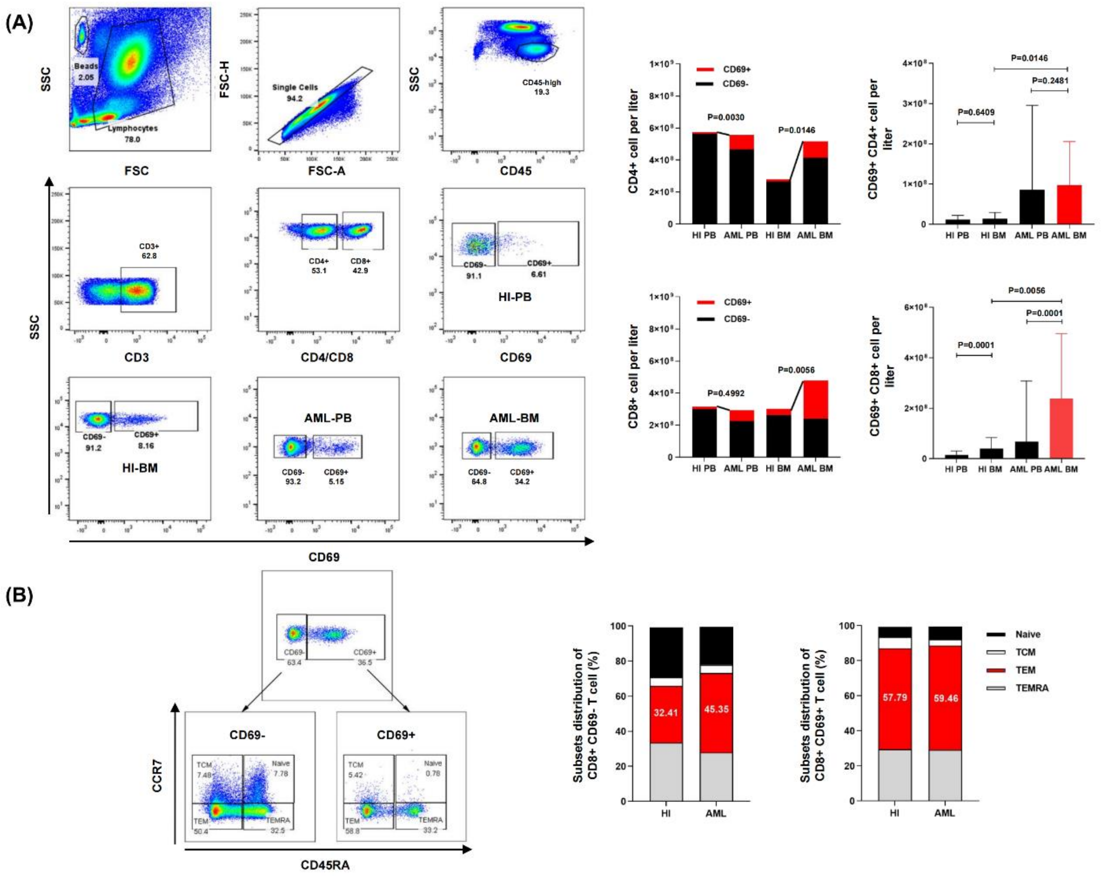

Figure S1. CD69 is mainly expressed by BM memory T cells and mainly distributed in the TEM subpopulation.

(A) Flow cytometry shows the gated logic and expression analysis of CD69 distribution in the PB and BM of HIs and patients with ND-AML. The histogram shows the absolute counts of $CD4^{+}CD69^{+}$ T cells and $CD8^{+}CD69^{+}$ T cells in the PB and BM of HIs and patients with AML. The number (n) of samples included in each subset analysis was as follows: $CD8^{+}CD69^{+}$: [HI: n(PB)=41, n(BM)=18, AML: n(PB)=29, n(BM)=25], $CD4^{+}CD69^{+}$: [HI: n(PB)=33, n(BM)=16, AML: n(PB)=29, n(BM)=23]

(B) Flow cytometry shows the subtype differentiation analysis and the corresponding absolute cell ratios of $CD69^{-}$ and $CD69^{+}$ in the BM of HIs and patients with ND-AML.

Paired t test was used for paired sample analysis. Mann–Whitney test was used for unpaired sample analysis. *, $p < 0.05$, **, $p < 0.01$, ***, $p < 0.001$, ****, $p < 0.0001$, ns: not significant.

Notes: PB, peripheral blood; BM, bone marrow; ND, newly diagnosed; and HI, healthy individual.

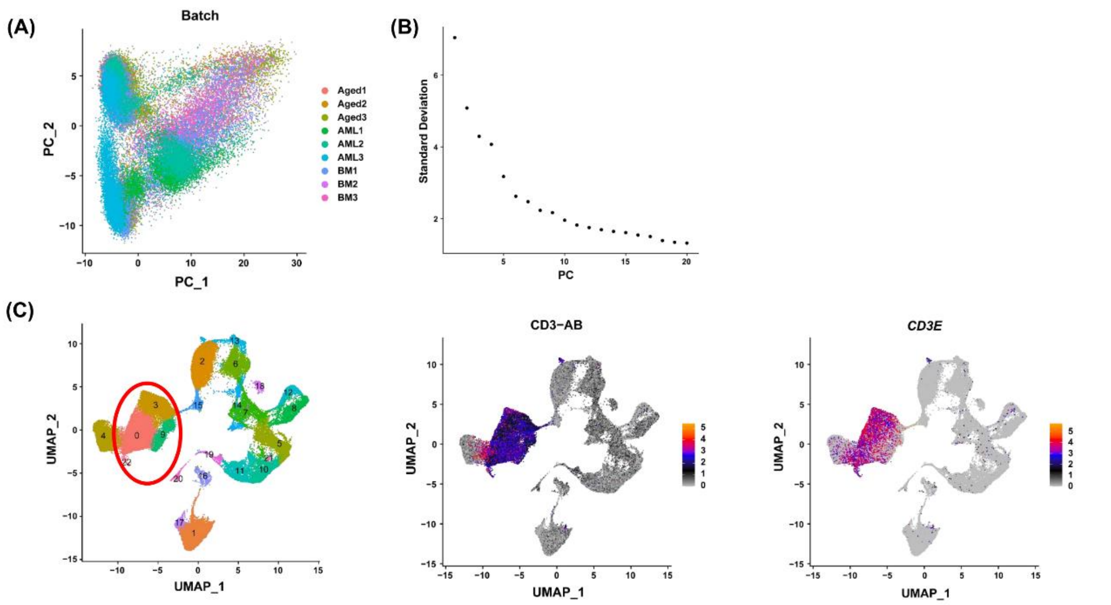

Figure S2. T cell subpopulation in single-cell proteogenomic dataset.

(A) PCA (Principal components analysis) visualization of single cells from BM. Single cells from different samples are depicted with distinct colors.

(B) Elbow plot used for identifying the optimal number of principal components (PCs).

(C) UMAP visualization of BM single cell clusters for single-cell proteogenomic dataset, subpopulation of T cell was highlighted by red cycle (left). Different cell types were depicted with distinct colors. Projection of CD3-AB and CD3E expression levels for single cells of BM（middle and right）.

2. Supplemental Tables

Table S1. Clinical information of ND-AML

Number	Age	Gender	OS	Status	FAB	*DNMT3*	*EVI1*	*IDH1/2*	*FLT3*	*NPM1*	*CEBPA*	Risk stratification	Chromosome	Treatment
P1	62.00	F	589.00	dead	M2a	*DNMT3-*	*EVI1-*	*IDH1/2-*	*FLT3+*	*NPM1-*	*CEBPA+*	Intermediate	unknown	Specific treatment unknown
P2	34.00	M	1152.00	alive	M2	*DNMT3-*	*EVI1-*	*IDH1/2-*	*FLT3-*	*NPM1-*	*CEBPA-*	Intermediate	AML with t (8;21) (q22;q22)	Cytarabine+Daunorubicin
P3	62.00	F	53.00	dead	M4	*DNMT3-*	*EVI1-*	*IDH1/2-*	*FLT3+*	*NPM1-*	*CEBPA-*	Adverse	AML with t (6;9)	Desoxycythromycin+Cytarabine
P4	16.00	M	212.00	alive	Unclassfied	*DNMT3-*	*EVI1-*	*IDH1/2-*	*FLT3+*	*NPM1+*	*CEBPA-*	Intermediate	AML with 46, XY	Cytarabine+Daunorubicin
P5	39.00	M	1125.00	alive	M2	*DNMT3-*	*EVI1-*	*IDH1/2-*	*FLT3-*	*NPM1-*	*CEBPA+*	Favorable	AML with 46, XY	Specific treatment unknown
P6	57.00	M	947.00	alive	M2	*DNMT3-*	*EVI1-*	*IDH1/2-*	*FLT3-*	*NPM1-*	*CEBPA+*	Intermediate	AML with 46, XY	Specific treatment unknown
P7	22.00	F	904.00	alive	Unclassfied	*DNMT3-*	*EVI1-*	*IDH1/2-*	*FLT3-*	*NPM1-*	*CEBPA+*	Favorable	AML with 46, XX	Cytarabine+Daunorubicin
P8	23.00	M	2.00	dead	M5	unknown	unknown	unknown	unknown	unknown	unknown	unknown	unknown	Specific treatment unknown
P9	68.00	M	178.00	dead	M4	*DNMT3-*	*EVI1-*	*IDH1/2-*	*FLT3-*	*NPM1-*	*CEBPA+*	Adverse	AML with 46, XY	Desoxycythromycin+Cytarabine
P10	76.00	M	88.00	dead	Unclassfied	*DNMT3-*	*EVI1-*	*IDH1/2-*	*FLT3-*	*NPM1-*	*CEBPA+*	Adverse	AML with 46, XY	Specific treatment unknown
P11	67.00	F	216.00	dead	M5a	*DNMT3-*	*EVI1-*	*IDH1/2-*	*FLT3-*	*NPM1-*	*CEBPA-*	Intermediate	AML with 47, XX, +8	Specific treatment unknown
P12	58.00	M	42.00	dead	Unclassfied	*DNMT3-*	*EVI1-*	*IDH1/2-*	*FLT3-*	*NPM1-*	*CEBPA-*	Adverse	AML with 46, XY, t (6;9) (p23;q34)/46, XY	Specific treatment unknown
P13	54.00	M	972.00	alive	M4/M5	*DNMT3-*	*EVI1+*	*IDH1/2-*	*FLT3+*	*NPM1-*	*CEBPA-*	Adverse	AML with 46, XY	Cytarabine+Venetoclax
P14	29.00	F	295.00	dead	M5b	*DNMT3+*	*EVI1-*	*IDH1/2-*	*FLT3+*	*NPM1+*	*CEBPA-*	Intermediate	AML with 46, XX	Specific treatment unknown
P15	70.00	M	19.00	dead	M2a	*DNMT3-*	*EVI1-*	*IDH1/2-*	*FLT3-*	*NPM1-*	*CEBPA-*	Adverse	AML with 44 X, -Y, add(10) (p14), -11/45, item, +mar	Cytarabine
P16	74.00	M	19.00	dead	M4/M5	*DNMT3-*	*EVI1-*	*IDH1/2-*	*FLT3-*	*NPM1-*	*CEBPA-*	Favorable	unknown	Decitabine
P17	27.00	F	852.00	alive	Unclassfied	unknown	unknown	unknown	unknown	unknown	unknown	unknown	unknown	Specific treatment unknown
P18	38.00	F	1159.00	alive	M2	*DNMT3-*	*EVI1-*	*IDH1/2-*	*FLT3-*	*NPM1-*	*CEBPA-*	Adverse	AML with -7/7q-	Specific treatment unknown
P19	67.00	M	296.00	alive	Unclassfied	*DNMT3-*	*EVI1-*	*IDH1/2-*	*FLT3+*	*NPM1+*	*CEBPA-*	Intermediate	unknown	Decitabine+Aclarubicin+Cytarabine
P20	14.00	F	1475.00	alive	M5b	*DNMT3-*	*EVI1-*	*IDH1/2-*	*FLT3-*	*NPM1-*	*CEBPA+*	Favorable	AML with 46, XX	Desoxycythromycin+Cytarabine
P21	53.00	F	90.00	dead	M2	*DNMT3-*	*EVI1-*	*IDH1/2-*	*FLT3-*	*NPM1-*	*CEBPA-*	Adverse	AML with 46, XY, del(5) (q13q33)/46,XY	Specific treatment unknown
P22	76.00	M	17.00	dead	Unclassfied	unknown	unknown	unknown	unknown	unknown	unknown	Intermediate	AML with 46, XY/47, XY +11	Specific treatment unknown
P23	66.00	F	452.00	alive	M2a	*DNMT3-*	*EVI1-*	*IDH1/2-*	*FLT3-*	*NPM1+*	*CEBPA-*	Favorable	unknown	Specific treatment unknown
P24	26.00	F	175.00	dead	M2	*DNMT3+*	*EVI1+*	*IDH1/2-*	*FLT3+*	*NPM1-*	*CEBPA-*	Adverse	AML with 45, XX, -7	Desoxycythromycin+Cytarabine
P25	21.00	M	885.00	alive	M5	*DNMT3-*	*EVI1-*	*IDH1/2-*	*FLT3+*	*NPM1-*	*CEBPA-*	Adverse	AML with 46, XY	Specific treatment unknown
P26	46.00	F	222.00	dead	M5	*DNMT3-*	*EVI1-*	*IDH1/2-*	*FLT3-*	*NPM1-*	*CEBPA-*	Intermediate	AML with t (11;22) (q23;q11.2)	Idarubicin+Cytarabine
P27	72.00	M	592.00	alive	M5b	*DNMT3-*	*EVI1+*	*IDH1/2-*	*FLT3-*	*NPM1-*	*CEBPA-*	Adverse	AML with 46, XY del(9) (q13q32)/46, XY	Decitabine+Aclarubicin+Cytarabine
P28	30.00	M	1656.00	alive	M2	*DNMT3-*	*EVI1-*	*IDH1/2-*	*FLT3-*	*NPM1-*	*CEBPA+*	Intermediate	AML with 46, XY, 10; 45, XY, -18, 4 and aneuploidy 6	Cytarabine+Daunorubicin
P29	35.00	F	485.00	dead	M2	*DNMT3+*	*EVI1-*	*IDH1/2+*	*FLT3+*	*NPM1-*	*CEBPA+*	Adverse	AML with 45, XX, -7	Desoxycythromycin+Cytarabine
P30	70.00	F	933.00	alive	M5b	unknown	unknown	unknown	unknown	unknown	unknown	unknown	AML with 46, XX	Azacitidine+Venetoclax
P31	37.00	F	206.00	dead	M5	*DNMT3-*	*EVI1-*	*IDH1/2-*	*FLT3+*	*NPM1-*	*CEBPA+*	Intermediate	unknown	Desoxycythromycin+Cytarabine
P32	67.00	M	144.00	dead	M2	*DNMT3-*	*EVI1-*	*IDH1/2-*	*FLT3+*	*NPM1-*	*CEBPA+*	Intermediate	AML with 46, XY	No chemotherapy or hypomethylation therapy
P33	67.00	F	112.00	alive	M2	*DNMT3-*	*EVI1-*	*IDH1/2-*	*FLT3-*	*NPM1-*	*CEBPA-*	Adverse	AML with 46, XX	Mitoxantrone
P34	63.00	M	1608.00	alive	M4	*DNMT3-*	*EVI1-*	*IDH1/2-*	*FLT3-*	*NPM1-*	*CEBPA-*	Favorable	AML with 46, XY	Idarubicin+Azacitidine
P35	78.00	M	681.00	dead	Unclassfied	*DNMT3-*	*EVI1-*	*IDH1/2-*	*FLT3-*	*NPM1-*	*CEBPA-*	Intermediate	AML with 46, XY	Specific treatment unknown
P36	75.00	F	824.00	dead	M2	unknown	unknown	unknown	unknown	unknown	unknown	Favorable	unknown	Odisil+Aclarubicin+Cytarabine
P37	78.00	F	53.00	dead	M5b	*DNMT3-*	*EVI1-*	*IDH1/2-*	*FLT3-*	*NPM1-*	*CEBPA-*	Favorable	unknown	Specific treatment unknown
P38	80.00	M	334.00	dead	M4	*DNMT3-*	*EVI1+*	*IDH1/2-*	*FLT3-*	*NPM1-*	*CEBPA-*	Adverse	AML with 46, XY	Decitabine
P39	50.00	F	170.00	alive	M5b	unknown	unknown	unknown	unknown	unknown	unknown	Intermediate	AML with 47, XX, +8/46, XX	Cytarabine+Daunorubicin
P40	38.00	M	642.00	alive	M5	*DNMT3-*	*EVI1-*	*IDH1/2-*	*FLT3+*	*NPM1-*	*CEBPA+*	Intermediate	AML with 46, XY	Desoxycythromycin+Cytarabine
P41	63.00	M	297.00	dead	M2	*DNMT3-*	*EVI1-*	*IDH1/2-*	*FLT3+*	*NPM1-*	*CEBPA-*	Intermediate	unknown	Decitabine+Venetoclax
P42	35.00	F	1104.00	alive	M5b	unknown	unknown	unknown	unknown	unknown	unknown	Intermediate	unknown	Ainonin+Cytarabine

Table S2. The specific cut-off of $CD8^+$ TRM-like, $CD69^-$ TEM, and $CD69^+CD8^+$ T cells.

Classification	Low	Age (median)	High	Age (median)
**$CD8^+$ TRM-like/CD8**	1.73-25.77	38.5	26.25-58.29	65
**$CD69^-$ TEM/CD8**	3.77-20.93	55	21.15-66.71	60
**$CD69^+$/CD8**	3.05-15.90	34	16.20-81.80	63

Table S3. Choice of Kaplan–Meier plotter

**Aff id/Gene symbol (Use mean expression of selected genes)**	CD8A/CD69/TOX								
**Split patients by**	median, Auto select best cut off								
**Survival**	Overall survival (n=1608)								
**Follow up threshold**	60 months								
**Probe set options**	all probe sets per gene								
**Karyotype**	all								
**FAB M**	all	all	all	M0	M1	M2	M4	M5	M6
**Gender**	all								
**Sample source**	bone marrow (n=796)								
**Treatment**	all								
**Dataset**	all	GSE12417	GSE37642	all	all	all	all	all	all
**Number of patients**	787	235	552	28	178	242	171	90	31
**Restrict analysis to selected cohorts**	all								
